# Supplementary material for: Comprehensive Annotation and Functional Exploration of MicroRNAs in Lettuce
Source: Front Plant Sci. 2021 Dec 24;12:781836. doi: 10.3389/fpls.2021.781836 (PMC8739914; doi:10.3389/fpls.2021.781836)
Supplement: Supplementary file 2 [file Data_Sheet_8.ZIP › GSM2230748.plot/Lsa-miR172b_Lsat_1_v5_gn_5_2120.1_1175_TPlot.pdf]

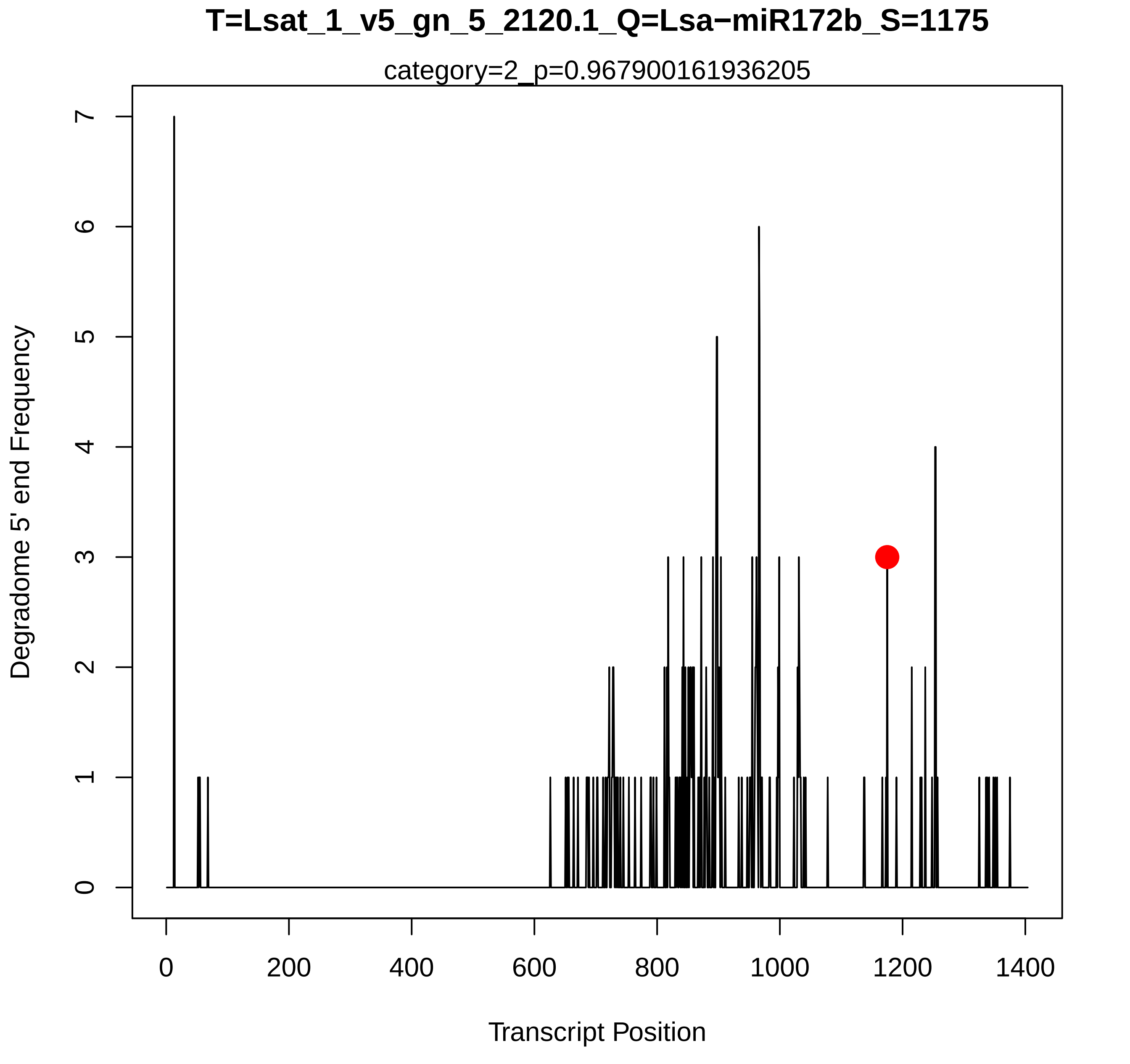
T=Lsat_1_v5_gn_5_2120.1_Q=Lsa−miR172b_S=1175
category=2_p=0.967900161936205
Degradome 5' end Frequency
Transcript Position
0
1
2
3
4
5
6
7
0
200
400
600
800
1000
1200
1400
